# Professional knowledge complements general technology acceptance in mathematics teachers’ critical behavioral intention toward AI

**DOI:** 10.3389/fpsyg.2026.1911467

**Published:** 2026-07-23

**Authors:** Chang Shu, Yangui Peng, Bo Wang, Hui Chen, Shengnan Bai

**Affiliations:** 1 School of Mathematics and Statistics, Northeast Normal University, Changchun, China; 2 School of Mathematics, Anshan Normal University, Anshan, China; 3 School of Mathematics and Statistics, Changchun University of Technology, Changchun, China; 4 School of Mathematics and Statistics, Henan University, Kaifeng, China

**Keywords:** mathematics teacher, critical behavioral intention, unified theory of acceptance and use of technology, mathematical knowledge for teaching, perceived trust

## Abstract

**Introduction:**

Artificial Intelligence (AI) is transforming education globally. In mathematics education, AI is being rapidly integrated, making teachers’ critical behavioral intention (CBI) toward AI increasingly essential. Mathematics teachers’ CBI refers to their ability to evaluate AI-generated outputs for mathematical accuracy and pedagogical appropriateness aligned with students’ Zone of Proximal Development (ZPD). Current technology acceptance models rarely incorporate subject-specific factors. This study extends the Unified Theory of Acceptance and Use of Technology (UTAUT) model by adding two core dimensions of Mathematical Knowledge for Teaching (MKT), specifically Specialized Content Knowledge (SCK) and Knowledge of Content and Students (KCS), with perceived trust (PT) as a mediator.

**Methods:**

A survey was administered to 169 mathematics teachers and PLS-SEM was employed to test models.

**Results:**

In the baseline UTAUT model, both performance expectancy (PE) and effort expectancy (EE) significantly predicted CBI. In the full model (integrating SCK, KCS and PT), only PE remained a significant direct effect. KCS significantly influenced PT, which in turn predicted CBI; SCK showed no such effect. The full model explained 56.3% of the variance in CBI.

**Discussion:**

To better harness AI in mathematics teaching, teacher professional development should ensure solid SCK while placing greater emphasis on KCS, and AI system design should strengthen PT rather than merely prioritize usability and operational efficiency.

## 1 Introduction

With the rapid progress of artificial intelligence (AI), the technology is being increasingly adopted in mathematics education. Applications like ChatGPT, DeepSeek, and Seewo Whiteboard create new opportunities for personalized and interactive learning, instant feedback, and the cultivation of students’ mathematical potential (Baidoo-Anu and Ansah, 2023; Almarashdi et al., 2024; Cheng, 2025; Pepin et al., 2025; Nikolic et al., 2026). However, many studies have shown that large language models (LLMs) present obvious reasoning limitations at all levels of mathematics, from primary school problems to International Mathematical Olympiad and advanced mathematics (Lo, 2023; Botana and Recio, 2024; Jiang et al., 2024; Spreitzer et al., 2024; Parra et al., 2026). Some studies have also indicated that LLMs do not ensure that the tutoring support is either valid or pedagogically appropriate, such as deviating from expected pedagogical strategies or leaking answers to students (Chowdhury et al., 2024). As Parra et al. (2026) points out, we need to carefully consider two risks of LLMs in mathematics education. First, because they lack genuine symbolic reasoning, they frequently cause significant mathematical errors. Second, they remain incapable of applying suitable teaching strategies. Therefore, whether AI can indeed play a positive and effective role in mathematics teaching largely depends on teachers’ willingness and ability to critically use such tools. For example, teachers need to evaluate AI outputs for mathematical accuracy, pedagogical appropriateness, and alignment with student thinking (Almarashdi et al., 2024; McGalliard and Otten, 2025; Pepin et al., 2025; Gómez Niño et al., 2026).

Unlike general technology acceptance, critical behavioral intention (CBI) involves a reflective, evaluative orientation, which is particularly important in the professional contexts of mathematics teaching. Specifically, CBI refers to a reflective and evaluative stance toward AI in mathematics, characterized by critically assessing AI outputs for mathematical accuracy and pedagogical appropriateness while maintaining independent judgment. Moreover, teachers cannot blindly trust AI, rather, they must adopt a critical stance, and their ability to do so depends on their own professional knowledge. According to Celik et al. (2026), whether pre-service teachers can formulate effective prompts with GenAI depends on their Intelligent-TPACK, a domain-specific knowledge construct. This suggests that professional knowledge shapes AI-mediated task performance. Goh et al. (2025) found that higher dependency on generative AI among students was significantly associated with increased cognitive failures and weakened critical thinking. This suggests that excessive reliance on AI can weaken the cognitive abilities essential for mathematics learning, such as logical reasoning and independent judgment. This finding serves as an important reminder for mathematics teachers that they must prioritize cultivating their own CBI, and then guide students to also develop their habit of using AI critically.

From this perspective, as noted by our research team, AI essentially maps the static body of human mathematical knowledge (e.g., published papers, textbooks, monographs) onto a dynamic knowledge system, akin to a functional mapping. In teaching and self-learning, static knowledge can be conceptualized as nodes in a grid; when using AI, teachers and students pose questions around these nodes, and AI progressively refines and completes their thinking. Consequently, in the AI era, rote memorization becomes less meaningful, whereas understanding, judgment, and logical reasoning become paramount. It is precisely in this dynamic process, moving from static knowledge nodes to active questioning and refinement, that students’ mathematical potential can be tapped. This further underscores why teachers’ critical behavioral intention is essential.

Research on technology acceptance has commonly applied models such as the Technology Acceptance Model (TAM; Davis, 1989; Davis et al., 1989), the Unified Theory of Acceptance and Use of Technology (UTAUT; Venkatesh et al., 2003) and its extensions (Venkatesh et al., 2016). UTAUT theorizes that users’ technology acceptance and their usage behavior are directly determined by four key predictors: performance expectancy (PE), effort expectancy (EE), social influence (SI), and facilitating conditions (FC). Recent studies have extended UTAUT to explore how teachers accept AI in diverse educational settings (Nguyen et al., 2025; Watted, 2025; Xue et al., 2025; Hao et al., 2026). Similarly, UTAUT has been used to examine how students use generative AI in learning contexts (Tian et al., 2024; Yakubu et al., 2025; Wang and Guo, 2026). Creely (2026) similarly warns that GenAI can produce “false mastery” where learners appear knowledgeable through reliance on AI results, yet fail to understand the basic principles, and that “AI dependency” may erode autonomous cognitive capacities, a risk that is particularly salient in teacher education.

Most studies applying UTAUT to AI in education have measured general behavioral intention, with little attention to critical behavioral intention. Moreover, they have yielded mixed results regarding the impact of PE, EE, SI, and FC on users’ behavioral intention (BI), suggesting that the standard predictors may not fully capture decision processes when professional judgment is involved (Wijaya et al., 2024a; Noureddine et al., 2025; Örnek et al., 2026). Nevertheless, numerous investigations within the technology acceptance literature confirm that BI exerts a strong positive influence on actual use behavior (UB). A review of the literature from the original TAM to UTAUT confirms that the link between BI and UB is a widely validated and inherited core theoretical assumption (Bazine, 2025). While the impact of PE, EE, SI, and FC on BI may differ across contexts, the predictive power of BI on UB remains robust. Meanwhile, because AI has not yet been widely adopted in routine mathematics classroom practice across most regions of China, this study focuses only on BI and leaves actual UB outside the framework.

In mathematics education, teachers’ Mathematical Knowledge for Teaching (MKT) is crucial when they engage with AI-generated content (Khalloufi-Mouha, 2024). Among the six dimensions of MKT (Ball et al., 2008), Specialized Content Knowledge (SCK) and Knowledge of Content and Students (KCS) are particularly relevant to the application of AI in teaching (Lee and Kim, 2024). SCK, as defined by Ball et al. (2008), represents the unique mathematical competencies demanded by the work of teaching, such as evaluating the validity of nonstandard solutions. Within this study’s setting, which centers on AI-supported instruction, teachers are required to apply this knowledge to judge the mathematical correctness and logical soundness of AI-generated outputs. KCS, according to Ball et al. (2008), refers to teachers’ understanding of the ways in which students think about specific mathematical content, enabling them to anticipate common misconceptions. In this study, we operationalize this knowledge as the capacity to judge whether content produced by AI is pedagogically appropriate for specific students, particularly whether it falls within their Zone of Proximal Development (ZPD; Vygotsky, 1978) and can be adapted to scaffold their understanding (Wood et al., 1976). SCK is a necessary baseline for teachers to detect AI-generated mathematical errors; without it, teachers cannot judge the correctness of AI outputs and thus cannot use AI responsibly. Theoretically, pedagogical content knowledge is built upon content knowledge (Shulman, 1986; Hill et al., 2008). However, once a basic level of SCK is achieved, further variations in SCK may not further differentiate teachers’ trust or intention to use AI critically, a pattern consistent with empirical findings that strong SCK does not automatically translate into strong KCS (Wilkie, 2014; Agathangelou and Charalambous, 2021). Instead, the ability to evaluate pedagogical appropriateness (KCS) may play a more discriminative role. This study therefore examines both SCK as a foundational competency and KCS as an advanced competency in shaping teachers’ CBI. Thus, CBI toward AI in mathematics teaching involves two distinct dimensions: (1) evaluating mathematical correctness (SCK); (2) evaluating pedagogical suitability within the ZPD (KCS).

However, few studies have integrated MKT into UTAUT. Although the value of SCK and KCS in mathematics education is well documented, it remains unclear how these two dimensions influence teachers’ perceived trust (PT) in AI and their subsequent intention to use AI in a critical way within a technology acceptance framework. Specifically, it is unknown whether SCK and KCS affect CBI through PT, and which dimension plays a more significant role. Furthermore, PT has consistently been confirmed as a major intervening mechanism in technology acceptance (Slade et al., 2015; Alkhowaiter, 2022; Abdelazim et al., 2025; Amin et al., 2025; Zhang et al., 2026), but its role in the context of AI for mathematics teaching, especially when combined with SCK and KCS, has not been systematically examined. This extension aligns with Feng and Carolus (2026), who argued in a systematic review that psychological dimensions, including cognitive and non-cognitive elements, must be integral to models explaining how teachers engage with AI.

To fill these research gaps, this study puts forward an extended version of the UTAUT framework that incorporates SCK and KCS as antecedents of perceived trust, and includes perceived trust as a mediator between KCS, SCK and CBI. The target population is primary and secondary school mathematics teachers in China. According to the developed model, the following hypotheses are proposed:

- H1. PE has a positive direct effect on CBI
- H2. EE has a positive direct effect on CBI
- H3. SI has a positive direct effect on CBI
- H4. FC has a positive direct effect on CBI
- H5. SCK has a positive direct effect on PT
- H6. KCS has a positive direct effect on PT
- H7. PT has a positive direct effect on CBI
- H8. PT mediates the effect of SCK on CBI
- H9. PT mediates the effect of KCS on CBI

**Figure 1:**
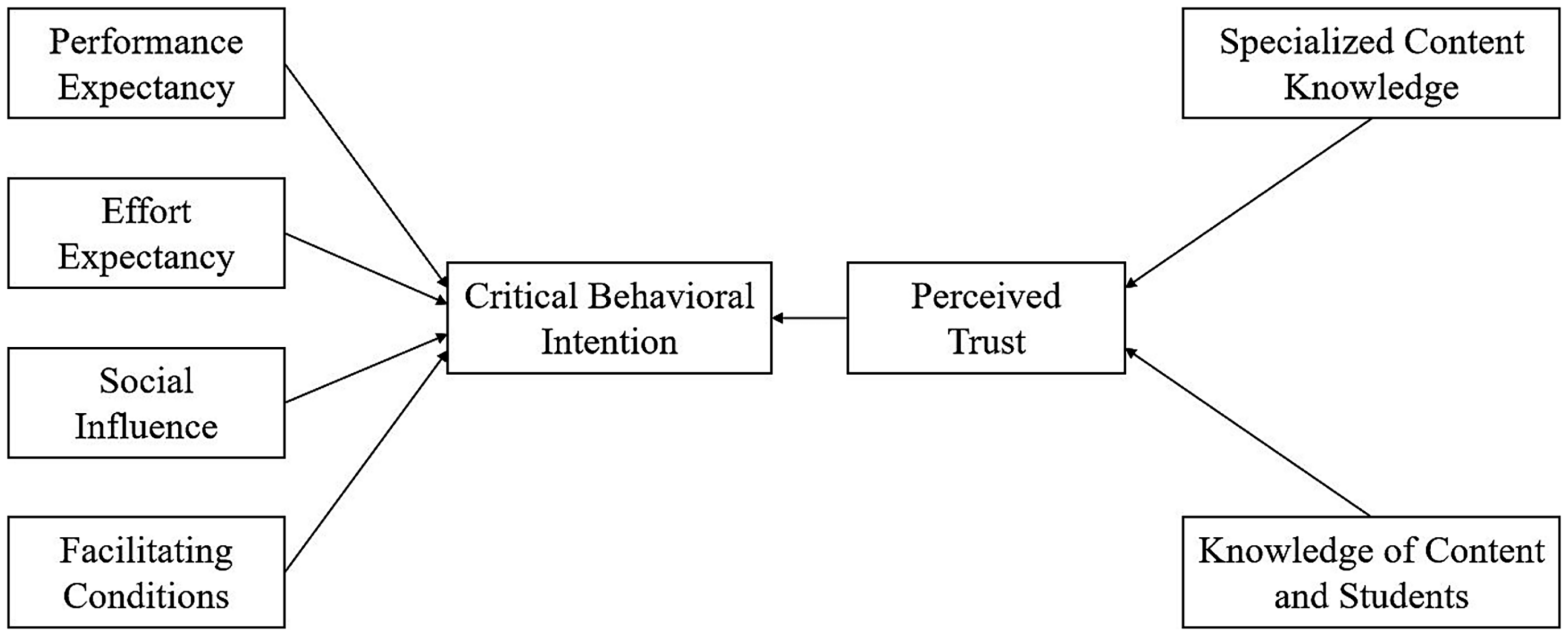
The conceptual model. The image is adapted from Venkatesh et al. (2003) and Mayer et al. (1995).

Drawing on the hypotheses formulated above, the conceptual model is presented in Figure 1.

The organization of the remainder of this paper is as follows. Section 2 is devoted to the materials and methods, including participants and procedure, measures, and analytical strategy. Section 3 presents the results. Section 4 discusses the findings, theoretical contributions, practical implications, limitations, and directions for future research. Section 5 concludes the study.

## 2 Materials and methods

### 2.1 Participants and procedure

This research employed a cross-sectional survey approach. The participants were primary and secondary school mathematics teachers in China, recruited through convenience sampling. The questionnaire was designed and distributed online using the Wenjuanxing platform. A link to the questionnaire was shared via the WeChat social platform in the spring of 2026, and the survey was open for one week. Eligible participants were currently employed mathematics teachers with at least a basic understanding of AI, such as ChatGPT, DeepSeek, and Seewo Whiteboard. No monetary compensation was provided, but a summary of the research findings was offered as an incentive.

The survey yielded 180 returned questionnaires. We excluded cases that met any of the following three criteria: (1) more than 5% missing data; (2) completion time less than 60 s; (3) clear patterned responses with a completion time of less than 90 s. A total of 169 valid responses were retained, with 93.9% of the returned questionnaires being valid. The cleaned dataset is available in the Supplementary Data Responses. The sample covered primary and secondary school mathematics teachers of different genders, teaching experiences, and educational backgrounds.

### 2.2 Measures

The questionnaire items were developed based on established scales from the English literature. Two bilingual researchers translated and adapted the items into Chinese. Specifically, the items for PE, EE, SI, FC, and CBI drew on those of Venkatesh et al. (2003); the items for SCK and KCS were adapted from the MKT framework of Ball et al. (2008); and the PT items were developed based on the trust theory of Mayer et al. (1995). All items were then adjusted to be appropriate for the context of AI-supported mathematics teaching. All items employed a six-point Likert scale, ranging from 1 (strongly disagree) to 6 (strongly agree), with no neutral option to prompt respondents toward a clear directional opinion. The final instrument comprised 24 items grouped into eight latent variables, each measured by three items. All items are presented in the Supplementary Data Questionnaire.

The following provides a brief definition and an example item for each construct:

- PE: The extent to which teachers perceive that AI enhances their mathematics instruction (e.g., “AI can improve my efficiency in mathematics teaching.”).
- EE: The extent to which teachers find AI easy to use and low in effort demand (e.g., “It is easy for me to learn to use AI for mathematics teaching.”).
- SI: The extent to which teachers perceive that colleagues, school leaders, and family members think they should use AI in their mathematics teaching (e.g., “My peers think I should use AI in mathematics teaching.”).
- FC: The extent to which teachers believe that technical infrastructure, resources, and support are available to use AI in their mathematics teaching (e.g., “My school provides AI for mathematics teaching.”).
- SCK: The ability to identify mathematical errors and evaluate the correctness of AI-generated mathematical content (e.g., “I can identify potential errors or inaccuracies in mathematical content output by AI.”).
- KCS: The ability to understand student thinking, anticipate common misconceptions, and adapt AI-generated explanations to support student learning which is essential for aligning AI outputs with students’ ZPD (e.g., “I can anticipate common confusions or misunderstandings that students may encounter when learning a mathematical concept.”).
- PT: The degree to which teachers trust the reliability and competence of AI-generated mathematical content (e.g., “I believe AI can generate reliable mathematical teaching content.”).
- CBI: The intention to use AI in a reflective, evaluative manner, maintaining independent judgment rather than uncritically accepting AI outputs (e.g., “I plan to use AI as a supplementary tool while maintaining my own independent judgment in mathematics teaching.”).

Before the formal survey, we invited 30 mathematics teachers to participate in a pilot test to examine the clarity, wording, and face validity of the items. The final sample did not contain the pilot test respondents. The reliability and validity of the final scales were satisfactory, as reported in the Results section.

### 2.3 Analytical strategy

Data were analyzed using Partial Least Squares Structural Equation Modeling (PLS-SEM) with SmartPLS version 4.0. This method was chosen over CB-SEM (e.g., using AMOS) because the primary aim of this study is exploratory. It seeks to explain mathematics teachers’ CBI and identify its key drivers, rather than to test a mature theory. Moreover, PLS-SEM is well suited for the current sample size, which is sufficient for stable estimation. It also does not require multivariate normality, which is rarely met by Likert scale data. Therefore, PLS-SEM is an appropriate analytical method for this study. Following the two-step procedure proposed by Hair et al. (2019), we first assessed the measurement model and then the structural model.

Measurement model assessment included:

- Indicator reliability: outer loadings of all items should exceed 0.708 (Hair et al., 2019).
- Internal consistency reliability: Cronbach’s α and composite reliability (CR) should exceed 0.70 (Hair et al., 2019).
- Convergent validity: average variance extracted (AVE) should exceed 0.50 (Hair et al., 2019).
- Discriminant validity: assessed using the heterotrait-monotrait ratio of correlations (HTMT) criterion, with a threshold of 0.90 (Hair et al., 2019).
- Collinearity diagnostics: the variance inflation factor (VIF) was used to examine multicollinearity among predictor constructs, with values below 5 indicating no critical collinearity (Hair et al., 2019).

Structural model assessment included:

- Path coefficients ( β ) and their significance: calculated using bootstrapping with 5,000 subsamples (Hair et al., 2019).
- Coefficients of determination ( $R^{2}$ ): for endogenous constructs, indicating the explanatory power of the model, with values of 0.25, 0.50, and 0.75 indicate weak, moderate, and substantial explanatory power, respectively (Hair et al., 2019).
- Predictive relevance ( $Q^{2}$ ): the $Q^{2}$ predict values were obtained via the PLS predict procedure. $Q^{2}$ predict values greater than 0 indicate that the model predictions outperform the naive mean prediction, confirming the model’s predictive relevance (Shmueli et al., 2016).
- Standardized root mean square residual (SRMR): while Hair et al. (2019) do not include a global goodness-of-fit measure in their core PLS-SEM evaluation framework, we also assessed the SRMR to provide a supplementary assessment of overall model fit. This approach follows the recommendation of Henseler et al. (2014), who introduced SRMR as a global fit measure for PLS path models to detect potential model misspecification. Values below 0.08 indicating good fit (Hu and Bentler, 1999) and below 0.10 acceptable fit (Henseler et al., 2014).

To test the mediation effects (SCK → PT → CBI and KCS → PT → CBI), we examined the bias-corrected bootstrap confidence intervals for the indirect effects. If the interval did not contain 0, the indirect effect was considered statistically significant (Preacher and Hayes, 2008).

All reported *p*-values are based on two-tailed tests.

## 3 Results

### 3.1 Descriptive statistics and measurement model

#### 3.1.1 Indicator reliability and internal consistency reliability

As shown in Table 1, all measurement items exceeded the 0.708 threshold for outer loadings, with values ranging from 0.788 to 0.942, indicating satisfactory indicator reliability. All constructs achieved Cronbach’s *α* and CR values exceeding 0.70, indicating satisfactory internal consistency.

**Table 1:** Outer loadings, Cronbach’s α , CR, and AVE.

Construct	Item	Outer loadings	Cronbach’s α	CR	AVE
PE	Q1	0.926	0.925	0.952	0.869
	Q2	0.942			
	Q3	0.929			
EE	Q4	0.809	0.802	0.883	0.715
	Q5	0.845			
	Q6	0.881			
SI	Q7	0.894	0.873	0.922	0.797
	Q8	0.863			
	Q9	0.921			
FC	Q10	0.928	0.883	0.928	0.811
	Q11	0.897			
	Q12	0.877			
SCK	Q13	0.788	0.836	0.900	0.751
	Q14	0.923			
	Q15	0.882			
KCS	Q16	0.898	0.875	0.923	0.800
	Q17	0.910			
	Q18	0.875			
PT	Q19	0.839	0.841	0.903	0.757
	Q20	0.894			
	Q21	0.877			
CBI	Q22	0.905	0.859	0.914	0.780
	Q23	0.896			
	Q24	0.848			

CR = composite reliability; AVE = average variance extracted.

#### 3.1.2 Convergent validity and discriminant validity

AVE for each construct (Table 1) exceeded 0.70, well above the 0.50 threshold, confirming strong convergent validity.

To evaluate discriminant validity, we applied the HTMT criterion. Table 2 reports that the highest HTMT values was 0.861 (between PE and SI), which fell below the widely accepted cutoff of 0.90. These results indicate that each construct in this study is empirically distinguishable from the rest.

**Table 2:** HTMT matrix.

Construct	PE	EE	SI	FC	SCK	KCS	PT
PE	–						
EE	0.816	–					
SI	0.861	0.838	–				
FC	0.701	0.816	0.854	–			
SCK	0.545	0.706	0.632	0.664	–		
KCS	0.699	0.772	0.763	0.734	0.793	–	
PT	0.772	0.805	0.796	0.697	0.635	0.849	–

All HTMT values are below 0.90, indicating adequate discriminant validity.

### 3.2 Structural model and hypothesis testing

#### 3.2.1 Baseline model

To provide a baseline for model comparison, we first estimated a baseline model that included only the four UTAUT constructs variables (PE, EE, SI, FC) as direct predictors of CBI. This model did not include the MKT dimensions (SCK, KCS) or PT. All four latent variables were simultaneously incorporated into the model and estimated using the PLS-SEM. The results (Table 3) showed that PE and EE had significant positive effects on CBI (PE: β = 0.344, *p* = 0.003; EE: β = 0.203, *p* = 0.029), whereas the path coefficients for SI and FC were not significant (SI: β = 0.144, *p* = 0.298; FC: β = 0.046, *p* = 0.702). The baseline model explained 44.3% of the variance in CBI ( $R^{2}$ = 0.443).

**Table 3:** Path coefficients of the baseline model.

Path	β	*p*-value	Significance
PE → CBI	0.344	0.003	**
EE → CBI	0.203	0.029	*
SI → CBI	0.144	0.298	ns
FC → CBI	0.046	0.702	ns

ns = not significant; *p < 0.05; **p < 0.01.

#### 3.2.2 Full model

**Figure 2:**
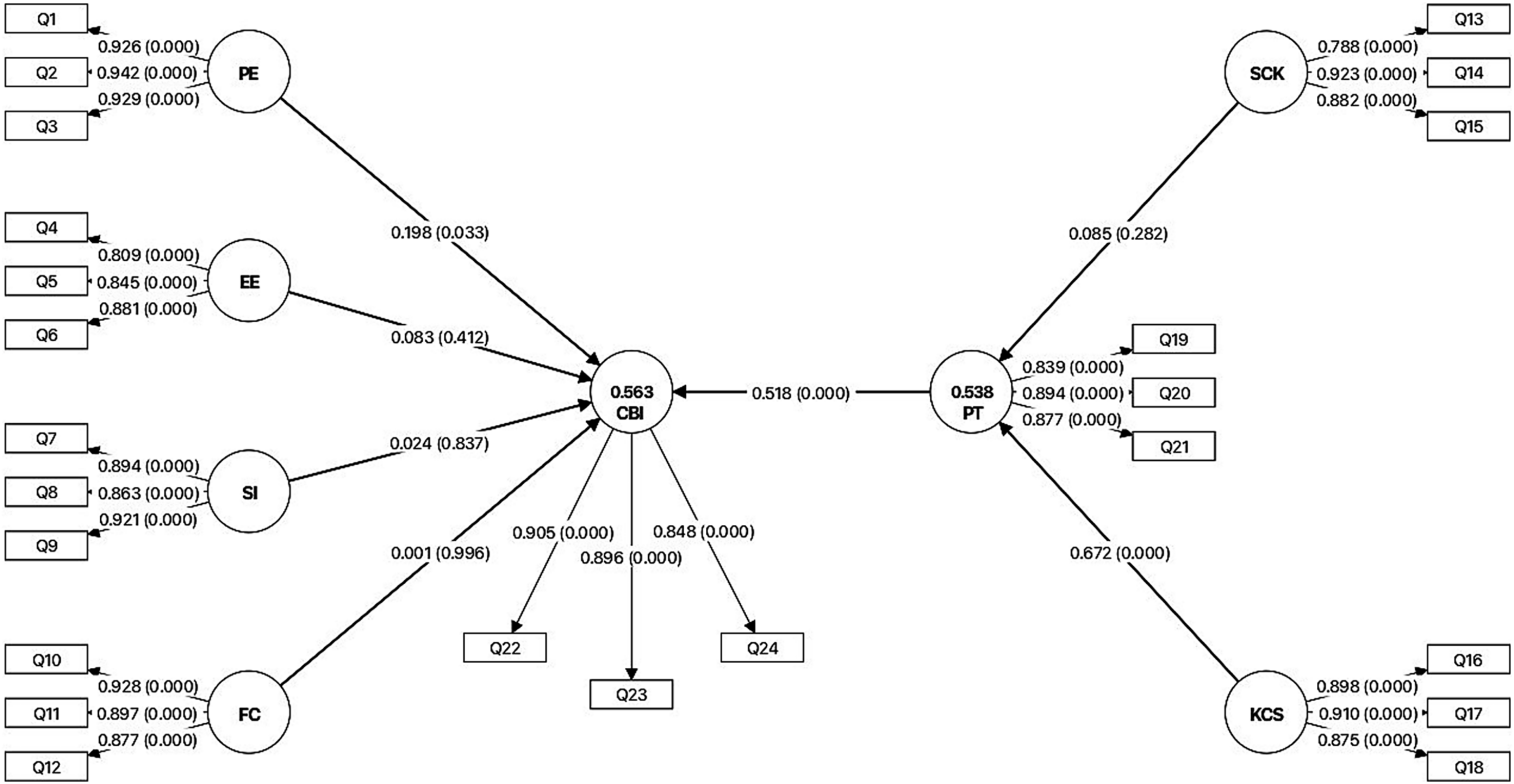
The full model with path coefficients and *p* values. Venkatesh et al. (2003) and Mayer et al. (1995).

The full PLS-SEM model estimated in this study is presented in Figure 2.

We then estimated the full structural model, which added the MKT dimensions (SCK and KCS) and PT to the baseline model. SCK and KCS served as antecedents of PT, PT served as a predictor of CBI, while retaining the four direct paths from the UTAUT variables (PE, EE, SI, FC) to CBI.

Before estimating the structural paths, we evaluated multicollinearity among the predictor constructs through the VIF. All VIF values remained under the threshold of 5, and they varied between 1.920 and 3.768. This indicates good data quality and no substantial multicollinearity, thus, the structural model estimates are reliable (Hair et al., 2019).

Table 4 presents the direct effects of the full model. PE had a significant positive effect on CBI ( β = 0.198, *p* = 0.033), supporting H1. In contrast, EE ( β = 0.083, *p* = 0.412), SI ( β = 0.024, *p* = 0.837), and FC ( β = 0.001, *p* = 0.996) did not significantly influence CBI; thus H2, H3, H4 were not supported. Regarding MKT, KCS significantly and positively predicted PT ( β = 0.672, *p* < 0.001), supporting H6, whereas SCK did not significantly predict PT ( β = 0.085, *p* = 0.282), failing to support H5. PT had a strong positive effect on CBI ( β = 0.518, *p* < 0.001), supporting H7.

**Table 4:** Direct effects of the full model.

Hypothesis	Path	β	*p*-value	Supported
H1	PE → CBI	0.198	0.033	Yes
H2	EE → CBI	0.083	0.412	No
H3	SI → CBI	0.024	0.837	No
H4	FC → CBI	0.001	0.996	No
H5	SCK → PT	0.085	0.282	No
H6	KCS → PT	0.672	<0.001	Yes
H7	PT → CBI	0.518	<0.001	Yes

Based on bias-corrected bootstrap with 5,000 subsamples.

Table 5 presents the specific mediation effects. The mediation effect of KCS on CBI via PT was significant (mediation effect = 0.348, *p* < 0.001, 95% CI [0.195, 0.524]), supporting H9. However, the mediation effect of SCK via PT was not significant (mediation effect = 0.044, *p* = 0.285, 95% CI [−0.034, 0.126]).

**Table 5:** Specific indirect effects of the full model.

Hypothesis	Path	Mediation effect	*p*-value	95%CI	Supported
H8	SCK → PT → CBI	0.044	0.285	[−0.034, 0.126]	No
H9	KCS → PT → CBI	0.348	<0.001	[0.195, 0.524]	Yes

Based on bias-corrected bootstrap with 5,000 subsamples.

The model explained 53.8% of the variance in PT ( $R^{2}$ = 0.538) and 56.3% of the variance in CBI ( $R^{2}$ = 0.563), indicating satisfactory explanatory power.

The PLS predict results showed that all indicator level $Q^{2}$ predict values for the two endogenous latent variables were positive, ranging from 0.322 to 0.433, indicating that the model has satisfactory predictive relevance.

Model fit was acceptable, with SRMR = 0.086, which is below the less conservative threshold of 0.10.

## 4 Discussion

The present study sought to integrate the UTAUT model, the SCK and KCS dimensions of the MKT framework, and PT to investigate mathematics teachers’ CBI toward using AI in mathematics teaching. Based on PLS-SEM analysis of 169 valid responses from primary and secondary school mathematics teachers, the main findings are as follows.

### 4.1 Summary of key findings

First, in the baseline model, both PE and EE had significant positive effects on CBI. However, in the full model that incorporated SCK, KCS, and PT, only PE retained a significant direct effect ( β = 0.198, *p* = 0.033), whereas the direct effects of EE, SI, and FC were all non-significant, with EE having lost its initial significance. This indicates that when mathematics teachers’ professional knowledge and trust mechanisms are taken into account, the role of traditional UTAUT predictors changes.

Second, of the two MKT dimensions, only KCS significantly and positively influenced PT ( β = 0.672, *p* < 0.001) and thereby exerted a significant mediation effect on CBI via PT (mediation effect = 0.348, *p* < 0.001). PT itself had a very strong direct positive effect on CBI ( β = 0.518, *p* < 0.001). SCK did not significantly predict PT, and consequently its mediation effect was also non-significant. The non-significant effect of SCK on PT does not imply that SCK is unimportant. Rather, it suggests that for the primary and secondary mathematics teachers in our sample, most possessed a sufficient level of SCK to evaluate the correctness of AI-generated content at the primary and secondary level, leaving little individual variation to explain differences in PT or CBI. This finding indicates that once this baseline level of SCK is satisfied, teachers’ trust and intention are primarily driven by their ability to adapt AI outputs to students’ learning needs, which is a higher order competency that exhibits greater individual differences (Rowland et al., 2005; Wilkie, 2014; Agathangelou and Charalambous, 2021; de Haan et al., 2024). These results reveal the crucial role of KCS in building trust in AI, while SCK alone is insufficient. From a cognitive load perspective (Sweller, 1988; Sweller et al., 2019), evaluating the pedagogical appropriateness of AI outputs within students’ ZPD imposes greater germane cognitive load than merely verifying mathematical correctness. As Creely (2026) notes, GenAI tasks that demand simultaneous attention to domain knowledge, pedagogy, and tool management can rapidly increase extraneous load; only teachers with well-developed KCS can manage this effectively. This may explain why KCS, but not SCK, significantly differentiated PT and CBI in our sample.

Third, the full model demonstrated satisfactory explanatory power ( $R^{2}$ = 0.538 for PT, 0.563 for CBI), adequate predictive relevance ( $Q^{2}$ = 0.392 for PT, 0.367 for CBI), and acceptable model fit (SRMR = 0.086). Overall, these indices indicate that the model performs well across all key evaluation criteria.

In summary, among the UTAUT factors, EE, SI, and FC do not directly drive mathematics teachers’ CBI; instead, CBI relies mainly on PE and the trust mechanism built by KCS. This finding underscores the central role of professional pedagogical knowledge for integrating AI into mathematics education.

### 4.2 Theoretical contributions

This study makes 3 significant theoretical contributions to the existing literature.

First, this study defines mathematics teachers’ critical behavioral intention as a construct distinct from general behavioral intention, emphasizing the evaluative and reflective stance required in the professional context of mathematics teaching. CBI encompasses two dimensions: correctness verification, which draws on SCK, and pedagogical appropriateness, which draws on KCS. General behavioral intention reflects only the strength of willingness to use, whereas CBI requires prudent evaluation and independent judgment. This distinction is particularly important given the high accuracy and logical rigor demanded in mathematics teaching. Through specific measurement items, this study operationalized CBI as a quantifiable construct and confirmed its discriminant validity from UTAUT dimensions via the HTMT criterion, providing a conceptual foundation for future research on critical technology use in professional education contexts.

Second, this study reveals that KCS, not SCK, is the key driver of teachers’ PT in AI and subsequent CBI. This finding suggests that situated trust in AI is built not merely on whether the outputs are mathematically correct, but on whether the teacher can judge if the output fits students’ ZPD. KCS enables teachers to anticipate misconceptions, interpret student thinking, and adapt AI-generated explanations, which are precisely the skills needed to scaffold learning within students’ ZPD, thereby tapping into students’ mathematical potential. SCK, while necessary as a baseline, does not further differentiate trust because most teachers in our sample possessed sufficient ability to judge correctness. Importantly, the nature of trust differs depending on teachers’ KCS level. Teachers with high KCS tend to develop informed, conditional trust, a reflective stance that underpins CBI. In contrast, teachers with low KCS either lack trust altogether or fall into blind, unconditional trust, accepting AI outputs without professional scrutiny. This view of trust is empirically supported by recent studies. Wijaya et al.’s (2024b) latent profile analysis of mathematics teachers, which revealed distinct patterns regarding AI literacy as well as trust, ranging from a “skeptical of AI” profile to a more dependent, less critical profile. Paiwithayasiritham et al.’s (2026) study on pre-service teachers showed that trust in AI depends more on perceived cognitive load reduction and responsible use than on perceived ease of use or efficacy, while also revealing that greater AI knowledge is associated with lower trust. Thus, KCS is the advanced competency that shapes critical, conditional trust, whereas SCK serves as the foundational baseline without further discriminating trust types.

Third, this study clarifies a boundary condition of the UTAUT model. The importance of extending conventional technology acceptance models for AI has been emphasized by Lelescu et al. (2025), who systematically reviewed the literature on educators’ trust in generative AI and concluded that conventional TAM/UTAUT frameworks are inadequate because they overlook the pedagogical and institutional aspects of trust beyond mere technical acceptance. Our study responds to this call by integrating two of MKT dimensions and PT into an extended UTAUT model, thereby addressing the very gap identified in their review. After adding MKT dimensions and trust mechanisms, the direct effect of EE changed from significant to non-significant, while PE remained significant; SI and FC were never significant. This change indicates that the influence of EE is replaced by KCS and trust, which are internal cognitive factors. When mathematics teachers form situated trust in AI based on their own KCS, whether the AI is “easy to use” no longer becomes a key factor in determining their CBI. The persistent significance of PE shows that mathematics teachers’ core concern remains whether AI can improve teaching quality and efficiency. The robust direct effect of PE observed in our full model echoes the findings of Wijaya et al. (2024a), who reported that PE uniquely and significantly predicted Chinese pre-service mathematics teachers’ BI toward AI chatbots. This cross-study consistency suggests that PE is a dominant driver of intention across teacher populations, whereas other UTAUT and its extended constructs depend on the context. The consistent non-significance of SI and FC further confirms the professional autonomy of mathematics teachers, they do not rely on others’ influence or external resources to drive decisions but rely on their own professional judgment.

This finding introduces a key boundary condition to the UTAUT model: in contexts of high professional autonomy, internal cognitive factors such as professional knowledge and trust become dominant, while PE remains a stable direct driver; among external drivers, EE loses its direct effect because it is replaced, whereas SI and FC never had predictive power. This study revises the applicability of UTAUT in professional educational contexts and suggests that future research on similar high autonomy professional groups should pay more attention to internal cognitive variables than to traditional external predictors.

### 4.3 Practical implications

The above findings lead to four practical implications: mathematics teacher education, the role positioning of AI, the cultivation of mathematical reasoning and critical judgment abilities, and the development of a trustworthy technological environment.

First, teacher training should emphasize KCS development, helping teachers learn to evaluate whether AI-generated content lies within students’ ZPD and how to adapt it to scaffold understanding. At the same time, SCK should not be neglected, as it serves as the baseline that ensures teachers can detect mathematical errors before considering pedagogical appropriateness. A practical training sequence could be: (1) ensure teachers can reliably verify AI outputs for correctness (SCK), (2) then develop their ability to judge and adapt AI content for different students (KCS). This two-stage approach acknowledges the hierarchical nature of teacher knowledge development and respects the foundational role of subject knowledge, which, as Rowland et al. (2005) argued, provides the “foundation” for all teaching decisions and for the transformation of knowledge into pedagogical actions.

Second, AI should be positioned as an auxiliary and supportive role. This study shows that mathematics teachers’ trust in AI is based on their professional judgment rather than on an evident reliance on technology. This is consistent with recent studies that underscore the value of teachers’ professional judgment and AI literacy when using AI to develop instructional materials for diverse learners (Feyijimi et al., 2025). Meanwhile, research has also indicated that in educational contexts, it is appropriate to promote reflective, autonomy-supportive AI use, viewing AI as a learning aid, not a substitute (Wang and Guo, 2026). Therefore, schools and educational administrators should make it clear that AI can be used for literature searching and idea summarization, but not replace teachers’ leading and guiding role. Otherwise, students’ thinking quality will decline, leading to the blind use of technology and increasing the risk in mathematics education.

Third, cultivate the habit and ability to “ask questions and generate ideas.” The core of critical behavioral intention lies in human beings’ own thinking initiative. Based on the above auxiliary positioning of AI, teacher education should first strengthen teachers’ own judgment and questioning habits, thereby fostering students’ critical thinking abilities. In daily teaching, mathematics teachers can deliberately design tasks such as “checking AI answers” or “revising AI-generated content” to encourage students to jointly examine the reasonableness of AI outputs, thereby transforming the process of AI use into a teaching opportunity for deeper learning. Only when both teachers and students maintain the habits of active questioning and independent thinking can AI truly become a support for mathematics education. This also reminds policymakers that regardless of how AI develops, the cultivation of core competencies such as mathematical reasoning, problem solving, and logical proof should not be weakened.

Fourth, AI developers and schools should jointly build trustworthy tools and supportive environments. This study found that perceived trust is a key mediating variable driving critical use. Therefore, AI should be designed to enhance transparency and verifiability, for example by providing detailed reasoning steps, marking uncertainty, and allowing teachers to modify intermediate results. On the school side, in addition to providing technical equipment and network support, case studies, demonstration lessons, and peer sharing should be organized to help teachers establish prudent trust in AI in authentic contexts, rather than simply encouraging them to “use AI more often.”

### 4.4 Limitations and future research directions

#### 4.4.1 Limitations

This study has several limitations.

First, sample size and representativeness. This study collected 169 valid responses, which meets the recommended requirements for PLS-SEM. However, the sample did not allow for multi-group comparisons across different school levels.

Second, limitation of the cross-sectional study. This study employed a cross-sectional survey, which cannot establish causal relationships between variables. Although we hypothesized that KCS influences trust and thereby affects critical behavioral intention, the possibility of reverse causality cannot be ruled out. For example, using AI may enhance teachers’ KCS.

Third, common method bias. The data were entirely gathered via self-administered questionnaires, a method that may be susceptible to common method bias. We took several measures to control for this, including anonymous surveys, clear instructions, a six-point Likert scale, and the removal of invalid responses. Moreover, the discriminant validity tests indicated good distinctiveness among the constructs, which helps to reduce the extent of common method bias.

Fourth, external validity and cross-cultural applicability. The sample of this study consisted of mathematics teachers in China. Cultural background and educational systems may compromise the generalizability of the findings.

#### 4.4.2 Future research directions

To overcome these limitations, subsequent studies should use larger and more varied samples to enable multi-group comparisons across different regions, school levels, and teaching experiences. One should employ longitudinal or experimental designs to determine causality. Multisource data could further reduce common method bias. Moreover, cross-cultural validation of the proposed model across different countries or educational systems would make the findings more widely applicable.

In addition to the methodological extensions, the results of this research suggest several directions for future investigation in mathematics pedagogy. To use AI critically, mathematics teachers must cultivate their abilities in appraisal, judgment, and logical reasoning. This capacity entails both verifying mathematical correctness and judging pedagogical appropriateness within students’ ZPD. We propose that the integrated framework of “mathematical intelligence” and “Research-Type Education Model” developed by our research team offers a promising practical pathway for achieving this cultivation goal. The basic framework of the Research-Type Education Model has been detailed in a Chinese doctoral dissertation by Wang (2025). Our research team is currently extending this line of work and applying it to the professional development of mathematics teachers. Further empirical validation of the proposed model, particularly its effectiveness in enhancing teachers’ SCK, KCS, and PT in AI, represents a valuable avenue for subsequent studies.

## 5 Conclusion

This study integrated the UTAUT model, the SCK and KCS dimensions of MKT framework, and PT to reveal the core mechanism of mathematics teachers’ critical behavioral intention toward AI. The main conclusions are as follows.

First, after introducing mathematics teachers’ professional knowledge and trust mechanisms, EE changed from significant in the baseline model to non-significant in the full model, whereas PE maintained a significant direct effect throughout; SI and FC were never significant in either model. This indicates that, for the mathematics teachers in our sample, the effect of perceived simplicity of use is replaced by trust built on knowledge.

Second, among the two dimensions of MKT, KCS emerges as the key driver of trust because it enables teachers to align AI outputs with students’ ZPD and scaffold their learning; SCK, while necessary, does not further differentiate trust. This shows that mathematics teachers’ trust in AI is situated, depending on whether they can judge whether AI-generated content fits students’ cognitive needs within the ZPD.

Third, PT is the core mediating mechanism linking mathematics teachers’ professional knowledge to CBI. The model explained over 56% of the variance in CBI, confirming the central role of trust in technology acceptance in professional education.

This study extends UTAUT from “general behavioral intention” to the context of “critical behavioral intention,” and for the first time introduces MKT as a professional specific antecedent of trust into the UTAUT framework. Practically, teacher training should emphasize KCS development, and AI should be designed to build trust rather than merely demonstrate ease of use.

In conclusion, for primary and secondary school mathematics teachers’ CBI toward AI, PT based on KCS is the central mechanism. This suggests that in professional education contexts, technology use research should shift from “external driving factors” to “internal cognitive factors.”

## Data Availability

The original contributions presented in the study are included in the article/Supplementary material, further inquiries can be directed to the corresponding author.
