# Supplementary material for: Professional knowledge complements general technology acceptance in mathematics teachers’ critical behavioral intention toward AI
Source: Front Psychol. 2026 Jul 23;17:1911467. doi: 10.3389/fpsyg.2026.1911467 (PMC13443043; doi:10.3389/fpsyg.2026.1911467)
Supplement: Supplementary file 1 [file Table_1.DOCX]

# Supplementary Data Questionnaire (Questionnaire items used in this study)

Questionnaire on Mathematics Teachers’ Intention to Use AI in Mathematics Teaching

This questionnaire aims to understand mathematics teachers’ perceptions, trust, and critical use intention regarding the employment of AI (e.g., ChatGPT, DeepSeek, Doubao, school‑based AI, etc.) in mathematics teaching. Your responses will provide valuable insights for mathematics teacher education and technology integration.

This questionnaire is anonymous, and all data will be used solely for academic research. Your personal information will not be disclosed. Please respond to each statement by choosing your agreement level based on your true feelings and actual situation by selecting the appropriate option.

Response Scale: 1 = Strongly Disagree, 2 = Disagree, 3 = Slightly Disagree, 4 = Slightly Agree, 5 = Agree, 6 = Strongly Agree.

Q1. AI can improve my efficiency in mathematics teaching. (PE1)

Q2. AI can help me better explain mathematical content to students. (PE2)

Q3. AI can improve the quality of my mathematics teaching. (PE3)

Q4. It is easy for me to learn to use AI for mathematics teaching. (EE1)

Q5. I can skillfully use AI to accomplish my desired teaching tasks. (EE2)

Q6. Using AI for mathematics teaching is easy and convenient. (EE3)

Q7. My peers think I should use AI in mathematics teaching. (SI1)

Q8. My school encourages me to use AI for mathematics teaching. (SI2)

Q9. My family and friends also support my use of AI in mathematics teaching. (SI3)

Q10. My school provides AI for mathematics teaching. (FC1)

Q11. My school offers training or resources on the use of AI in mathematics teaching. (FC2)

Q12. When I encounter problems using AI for mathematics teaching, I can receive timely help. (FC3)

Q13. I can identify potential errors or inaccuracies in mathematical content output by AI. (SCK1)

Q14. When the mathematical content provided by AI contains gaps or ambiguities, I can identify the missing logic. (SCK2)

Q15. When AI gives incorrect information, I can point out the problem until the AI provides the correct answer. (SCK3)

Q16. I can anticipate common confusions or misunderstandings that students may encounter when learning a mathematical concept. (KCS1)

Q17. I can analyze the cognitive reasons behind students' mathematical errors. (KCS2)

Q18. I can adjust the content output by AI to facilitate learning based on my understanding of students’ mathematical knowledge. (KCS3)

Q19. I believe AI can generate reliable mathematical teaching content. (PT1)

Q20. I trust that the output of AI in mathematics teaching is reliable, but I will verify it. (PT2)

Q21. I am willing to trust AI‑generated mathematical content after prudent evaluation. (PT3)

Q22. I am willing to use AI in my future mathematics teaching, but I will first critically evaluate their output. (CBI1)

Q23. Before using AI‑generated mathematical teaching content, I will carefully examine its accuracy and appropriateness. (CBI2)

Q24. I plan to use AI as a supplementary tool while maintaining my own independent judgment in mathematics teaching. (CBI3)
